# Resonance frequency is not always stable over time and could be related to the inter-beat interval

**DOI:** 10.1038/s41598-021-87867-8

**Published:** 2021-04-16

**Authors:** Lluis Capdevila, Eva Parrado, Juan Ramos-Castro, Rafael Zapata-Lamana, Jaume F. Lalanza

**Affiliations:** 1 Departament of Basic Psychology, Universitat Autònoma de Barcelona (UAB), Bellaterra, Barcelona, Spain; 2 Sport Research Institute, Universitat Autònoma de Barcelona (UAB), Bellaterra, Barcelona, Spain; 3 Department of Electronic Engineering, Biomedical and Electronic Instrumentation Group, Universitat Politècnica de Catalunya, Barcelona, Spain; 4 Escuela de Educación, Universidad de Concepción, Los Ángeles, Chile; 5 Department of Psychology, UiT The Arctic University of Norway, Tromsø, Norway

**Keywords:** Physiology, Psychology, Biomarkers, Cardiology

## Abstract

Heart Rate Variability Biofeedback (HRVB) is based on breathing at an optimal rate (or resonance frequency, RF) corresponding to the respiratory sinus arrhythmia (RSA). Our aim is to check whether the RF is a stable factor and analyse the HRV parameters individually per each breathing rate, comparing it with free slow breathing. A sample of 21 participants were trained in a test–retest HRVB protocol. The results indicated that RF changed between Test and Retest sessions in 66.7% of participants. This instability could be related to the average of interbeat interval (IBI). HRV time domain parameters (SDNN and RMSSD) were significantly higher for RF than for other breathing rates, including 6 breath/min and free slow breathing. Free slow breathing showed a lower heart rate averages than RF and other slow breathing rates. Overall, our study suggests the relevance of assessing RF individually and before each HRVB session, because the maximum cardiovascular benefits in terms of increasing HRV were found only at RF. Thus, breathing at the individualized and momentary frequency of resonance increases cardiac variability.

## Introduction

Heart Rate Variability Biofeedback (HRVB) (also called Resonance Frequency Biofeedback) is a relaxation technique based on the respiratory sinus arrhythmia (RSA), which is the variation in heart rate (HR) corresponding to breathing^1^. HRVB consists of a breathing training at the resonance frequency (RF), or optimal breathing at a frequency that produces maximal RSA.

Thus, its effectiveness would be based on RSA and the baroreflex (BR)^2,3^. HRVB is a highly promising intervention for improving health and life quality in patients of depression^4^ and other psychiatric disorders^5–8^, as well as pain^9^, asthma^10^, or pre-hypertension^11^. There is also evidence that HRVB is a good treatment for the non-clinical stressed population^5^, and it has been used to improve performance in elite athletes through enhancing psychophysiological variables^12^. A recent systematic review of the effects of HRVB concludes that their training improves the emotional health (anxiety, depression, anger), athletic and artistic performance, and to a lesser extent sleep and quality of life^13^. Notwithstanding this positive evidence, methodological studies are still needed for a better understanding of the variables involved in HRVB.

The most used protocol is based on the one from Lehrer et al.^1^. These authors presented the rationale and a manual for HRVB training. Based on previous studies, they talk about the “Two Closed-Loop” theory of baroreflex function to explain the effectiveness of biofeedback for respiratory sinus arrhythmia to produce resonance in the cardiovascular system. This first manual establishes the main procedure to be followed in the majority of subsequent research on HRVB. According to this procedure, in the first place, the resonant frequency of the participant is determined, and he/she is trained to breathe at his/her resonant frequency to produce maximal increases in amplitude of respiratory sinus arrythmia. Then, the participant is instructed to practice breathing at his/her own resonant frequency for several periods and times at home on your own^1^. Thus, in many subsequent investigations this procedure has been applied assuming that RF is stable over time. Typically, during HRVB training RF is assessed during sessions of HRVB and then the participant performs paced breathing always at this RF at home, assuming the stability of the RF. However, there are also studies that, instead of using this procedure, apply a predetermined breathing frequency, usually at 6 breaths/min (b/m) (e.g., Ref.^14^) or a progressive breathing system (e.g. Ref.^15^).

Steffen et al.^16^ demonstrated the relevance of an accurate detection of individual RF. HRVB at the RF increased HRV and mood in comparison to RF + 1b/m and a passive control group in a single 15 min session. This study demonstrated the relevance of breathing at the individual RF, although other rates or free slow breathing were not compared. In addition, since the HRVB technique was first proposed, it has been assumed that RF was stable over time if there was no specific training to change it. As far as we are aware, very few studies have examined the individual RF stability, or assessing the RF before each session^17,18^.

HRVB is a good method for controlling autonomic modulation^19^, and its training increases Heart Rate Variability (HRV)^20^, which is defined as the fluctuations in the time interval between consecutive beats^21^. In the scientific literature it is accepted that the greater the cardiac variability the better the general health, both physical and emotional, and that HRVB as a valid method to increase heart rate variability with beneficial effects^13^. For its part, HRV has been considered an index to assess the autonomic balance between the sympathetic and the parasympathetic system^22^. Indeed, HRV is considered an index of autonomic resilience, since it reflects the ability to recover from exposure to both physical and psychological stressors^23,24^. HRV parameters derived from time domain are based on the level of variability between the interbeat intervals (IBI). The most common variables are: (i) SDNN (or SDRR), the standard deviation of normal to normal IBI expressed in milliseconds (ms); and (ii) RMSSD, the root mean square of successive IBI differences expressed in ms^21^. RMSSD appears to be the most cost‐efficient measure of RSA^25^.

The aim of this study is to check whether the RF is a stable factor in a test–retest protocol. Furthermore, we want to analyse the HRV parameters individually per each breathing rate of the initial phase of the HRVB protocol. In this regard, we will compare RF with the mean of the other breathing rates, the breathing rate with the lowest RSA, the commonly used rate of 6  b/m, and with the free slow breathing. Hence, we could evaluate the relevance of checking the RF before each HRVB session or the effectivity, in terms of HRV increases, of following the pre-set breathing rate of 6.0 b/m.

## Methods

### Study design

**Figure 1:**
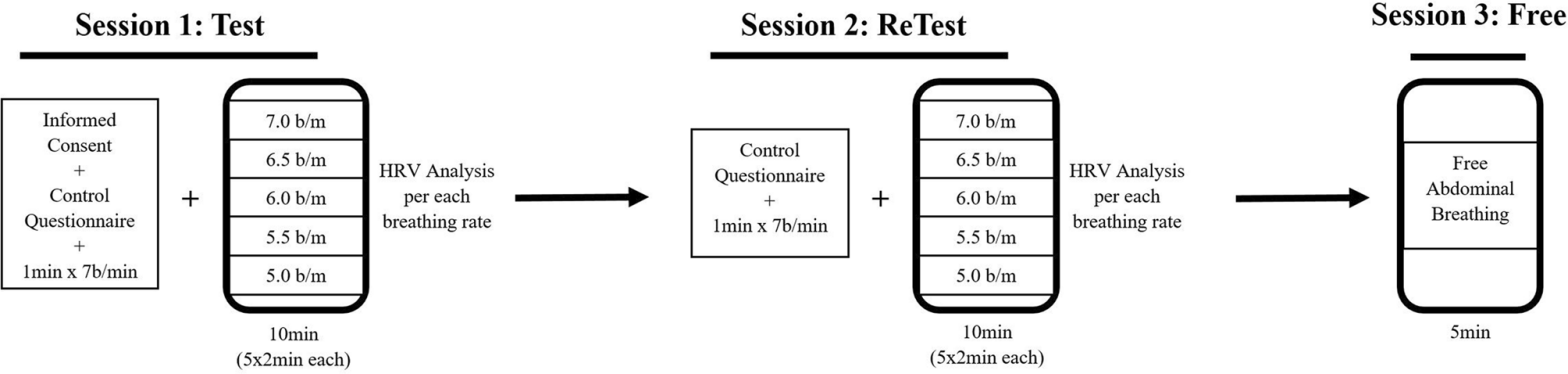
Schema of the study procedure.

The study consisted of three sessions with a two-cohort quasi-experimental design (Fig. 1). The RF was assessed during the first two sessions (Test and ReTest), and the HRV parameters were calculated for each breathing rate of the intitial phase of the HRVB protocol. In the third session, participants breathed abdominally and freely (without a metronome). Thus, comparisons were carried out between breathing rates and between sessions. All participants gave their informed consent, received information about the procedure, and were able to ask questions before starting the session, as well as being able to abandon the experiment at any time without giving any explanation. Experimental procedures were approved by the Ethics Committee of the Universitat Autònoma de Barcelona and the data were treated anonymously. All methods were performed in accordance with the relevant guidelines and regulations.

### Participants

The first cohort consisted of undergraduate university students, and the second cohort of administrative university staff. An e-mail was sent with the instructions, along with a link to a short ad hoc Google questionnaire with the exclusion criteria items and the experimental schedule options. The exclusion criteria included: being under 18 year and suffering for any cardiorespiratory disease. From the first cohort, 10 out of 14 participants completed all three experimental sessions with a correct heart rate signal (3 women and 7 men; mean age = 21.4 ± 1.6 years). One participant was also excluded from the analysis due to not following the assigned breathing rate in the Retest session. From the second cohort, 12 out of 20 university staff completed the three sessions breathing as instructed (10 women and 2 men; mean age = 49.3 ± 6.8 years). The loss of participants was due to cardiorespiratory diseases, a scheduled surgery, lack of an e-mail contact, and failure to complete the experimental sessions.

### Self-reported control measures

Participants were requested by e-mail 24–48 h before the experimental session to specifically avoid taking non-essential drugs 24 h before the experimental session, as well as to avoid high intensity physical activity or an unusual exercise 20 h before. They were also advised to avoid caffeine, smoking, or using any other psychostimulant 2 h before, as well as not to drink alcohol 10 h before, avoid heavy meals 3 h before, eating anything 1 h before, to sleep at least 6 h, and to wear comfortable clothes. An adaptation of a previous questionnaire^26^ was used to control those conditions of the participants just before each HRVB session. When participants attended the first lab session, they were asked to self-report their height.

### Heart rate variability

For the HRV analysis, participants wore a cardiac Polar Band H7 (Polar Electro, Finland). RR intervals with a resolution of 1 ms were sent by Bluetooth to the FitLabCoach App (Health and SportLab, Barcelona, Spain), which was downloaded into an iPad in order to record the RR series, analyse HRV parameters during the HRVB protocol. The accuracy and reliability of the Polar Band H7 was previously tested with the gold standard based on the ECG^26^. RR mean and time domain parameters (SDNN and RMSSD) were assessed. For the spectral analysis the IBI series were interpolated by a cubic spline to a sampling frequency of 4 Hz. We applied a Hanning window for each RR interval series for each breath frequency and the power spectrum was estimated by the FFT of the windowed series.

### Procedure for spectral estimation and RSA estimation

The error correction of RR series was based on outliers detection. The median of the last 10 RR intervals was used. The outliers were classified in false positives, false negatives or ectopic beats. A correction in the RR series was applied to keep the total duration of the recording^27^. After the artifact removal, the RR series were resampled to 4 Hz with a shape-preserving piecewise cubic interpolation for the spectral estimation. For each paced respiratory frequency interval, the corresponding RR interval was extracted from the whole RR resampled series. The segment was windowed with a Hann window and zero padded to a number of samples power of 2. The power spectrum was estimated by using an Fast Fourier Transform (FFT). The RSA was identified by looking for the maximum of the spectrum in a frequency interval ± 20 of the paced frequency. If a discrepancy greater than 5% was detected, we considered that the subject was not following the paced rhythm and the record was discarded.

### Heart rate variability biofeedback

The protocol designed by Lehrer et al.^1^ for HRVB was applied, with the modification of the breathing rates. Five breathing rates (7, 6.5, 6, 5.5, and 5 b/m; instead of 6.5–4.5) of 2 min each were used without a pause between them, and the seconds of inspiration and expiration were equal. The speed of the breathing rates was increased, as previous studies failed to find students breathing at 4.5 b/m and therefore we did not include it^16^. Moreover, in a sample of elite sport support staff, the optimal rate was between 6.5 and 5.5^17^, closer to 7 and far from 4.5 b/m. A sonorous metronome (like a human breathing sound), included in the FitLabCoach App and synchronized with the RR recording and HRV analysis (also with FitLabCoach App), was used as a pacer to guide participants during the HRVB. All HRV parameters were assessed for each breathing rate and compared among them. Therefore, HRV parameters were compared for the RF (the optimal rate or the highest RSA amplitude; based on the maximum amplitude from the spectral analysis, taking the higher value), the mean of other rates (excluding the RF), the lowest RSA amplitude (the worse rate; based on the maxim amplitude from the spectral analysis, but taking the lower value), and the 6 b/m rate (as it is the most chosen pre-set ratio in other studies) in the Test and Retest sessions. These categories of breathing rates were also compared to the HRV parameters obtained during the free slow breathing session. A parameter called RFE (resonance frequency expanded), which consists of taking the mean of HRV parameters of the nearest below and above rates from the RF (for example, RF = 6.5, RFE is the mean of 7.0, 6.5 and 6.0) was also compared with the RF.

### Procedure

Participants, in small groups, were scheduled in the laboratory for two (cohort 1) and three (cohort 2) consecutives weeks at the same hour and every 6–8 days. On arrival, participants were asked to sit down, read, and then accept and sign the informed consent and to complete the self-report control questionnaire. The participants then placed the cardiac chest band with a conductive gel drop to improve the electric contact between the electrodes and the skin and minimize detection errors in the RR recording. Participants were asked to sit normally in a relax position without crossing the legs, with the hands on the thighs and closing their eyes (to avoid visual interactions between participants and getting distracted). The researcher checked the position of each participant, since this could modify heart parameters^28^. The room was between 22 and 24 °C, with a natural, soft and indirect light. Before starting the HRVB, subjects breathed at 7 b/m for one minute following the sonorous metronome, in order to achieve the breathing rate and to become habituated to the breathing exercise. The Test session was ended after 10 min of HRVB. The retest (cohort 1) session was identical to the Test one, but adding a 5 min of seated and free slow breathing HRV assessment after the HRVB. For cohort 2, the Retest session followed the same protocol, but the free slow breathing was carried out one week later after another HRVB session. For the free slow breathing session, participants were instructed to breathe slow and deeply, in a “relaxing way”, but without indicating any breathing rate or breathing technique.

### Data analysis

HRV and HRVB data were obtained directly from the FitLabCoach App. A maximum signal error of 11% of RR intervals was accepted and filtered, although, the mean of signal error for all RR records was 0.91%. The error correction in the RR series and the HRV analysis was carried out with Matlab (MathWork, USA) scripts developed by the researches and validated in other publications^26,27^. The detection of the maximum RSA was done by looking for the highest power peak in the Fast Fourier Transformation of the RR series for all the breathing rates. We performed a Friedman test (non-parametric ANOVA for repeated measures) when we are comparing HRV parameters (IBImean -RRmean-, SDNN, RMSSD) between more than two breathing stages (RF, mean of the other breathing rates, breathing rate with the lowest RSA, rate of 6 b/m, and free slow breathing), due to the small sample size and the fact that some of the HRV parameters did not follow a normal distribution across the five breathing rates. Then, in case of significance, we calculated pairwise comparisons (Wilcoxon Test) with appropriate correction for multiple comparisons. Thus, Wilcoxon non-parametric analyses were carried out to compare the RF versus the mean of the other rates, the lowest, 6 b/m and RFE, and free breathing versus the RF, the mean of the other rates, the lowest and 6 b/m, only for the Retest session. We performed a mixed MANOVA 2 × 2 comparing the change of HRV parameters (IBImean; SDNN and RMSSD) between Test and Retest (within-subject factor) and comparing two RF groups (between-subject factor) according to: Group (1) Test–retest “RF Change” (14 participants); and Group (2) Test–retest “RF No-change” (7 participants). We performed correlation analysis (Spearman rho) independently for the two RF groups between HRV parameters and RF values in each session (Test and Retest). Results were expressed in terms of mean and standard deviation (SD) in tables, or standard error of the mean (SEM) in figures; *p* values < 0.05 were considered statistically significant.

## Results

### Self-reported control measures

The means of the self-reported heights were 176.5 cm (SD 3.92) for men and 163.42 cm (SD 5.92) for women (mean age 168.5, SD 8.40 for the total sample). There is no significant correlation between height and RF, neither for the test nor for the retest. In general, all participants followed the control conditions before the experimental sessions. From a total of 336 items of the control questionnaire (*8 control questions × 2 sessions × 21 participants*), 307 items were accomplished (91.4%), which means that conditions were similar between participants and for both sessions. Thereby, we could ensure that HRV parameters had not been altered by external factors, such as caffeine or tobacco.

### HRVB execution for test and retest sessions and RF calculation

Firstly, we checked that all participants followed the metronome during both Test and Retest sessions as has been explained in 2.5. Only one participant was excluded from the study for not following the breathing instructions. The mean of the actual breathing rates from the included participants are for the test and retest sessions: 6.90 (metronome: 7 b/m), 6.44 (metronome: 6.5 b/m), 5.91 (metronome: 6.0 b/m), 5.45 (metronome: 5.5 b/m) and 4.99 (metronome: 5.0 b/m).

**Figure 2:**
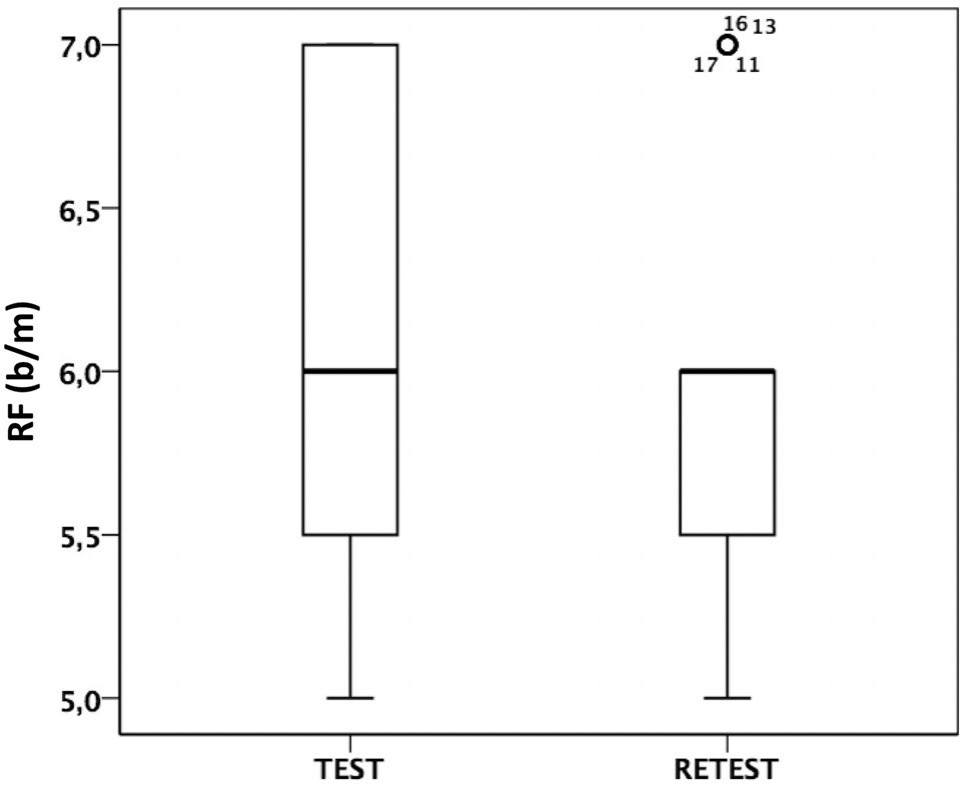
Comparative box plots of RF individual values (breaths per minute) at Test and Retest.

We observed some inconsistency in the RF between Test and Retest individually. Overall, the RF was similar between Test and Retest sessions, since the mean was 6.1 (SD 0.85; median 6) in Test and 5.9 (SD 5.9; median 6) in Retest session. Nevertheless, the RF changed between Test and Retest sessions in 66.7% of participants. Thus, 14 out of 21 subjects had a different RF during the retest compared to test. Nine subjects had a lower RF during retest and 5 subjects showed an increased RF during retest. Figure 2 represents the comparative box plots of RF individual values (in breaths per minute) at Test and Retest, showing a different distribution in both sessions.

**Figure 3:**
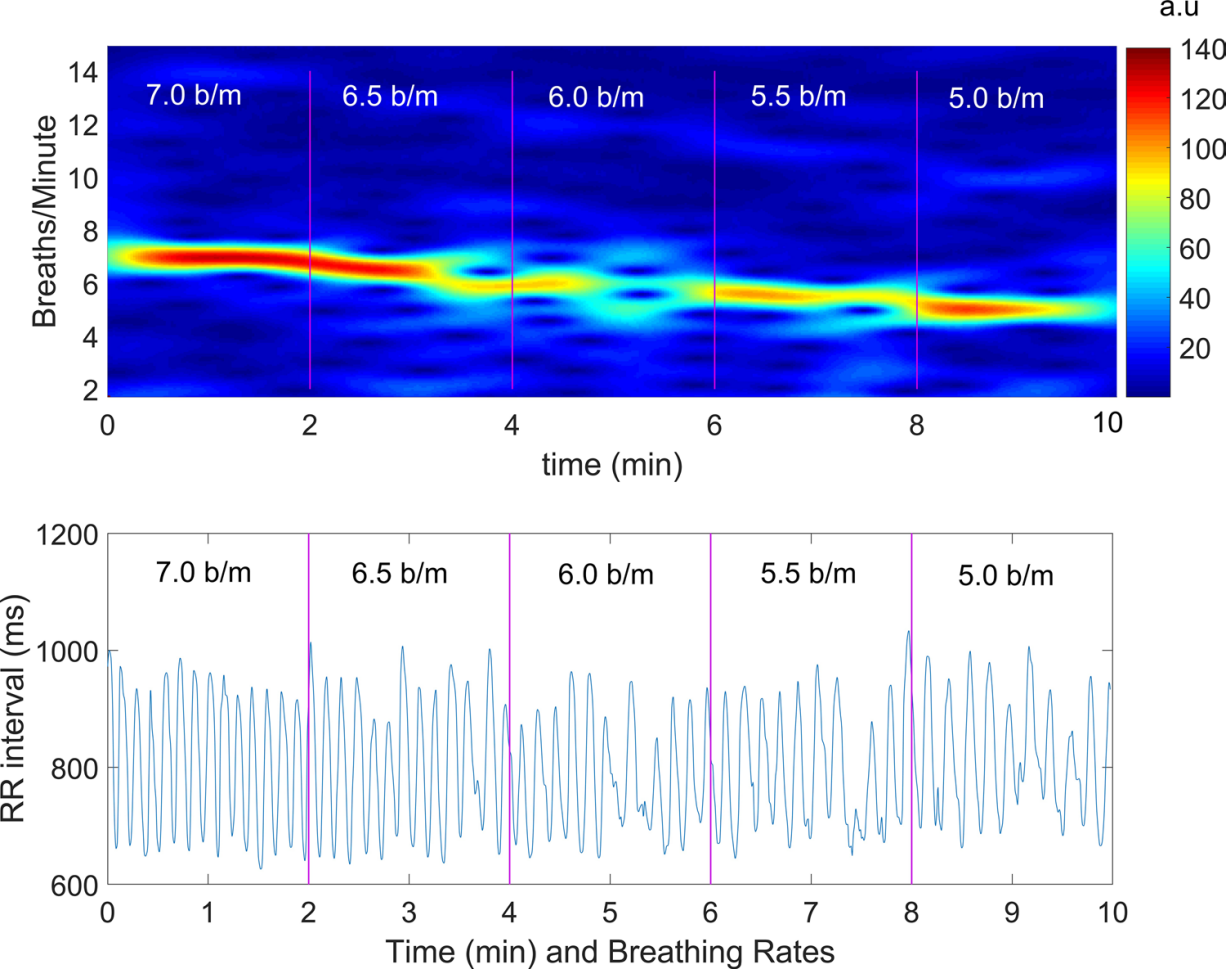
Representation of the spectrogram analysis (STFT, 120 s window, 1.5 s resolution, Hann window) of the IBI series (bottom trace) for each breathing rate (7, 6.5, 6, 5.5, 5 b/m) during the HRVB of Participant 15 during the Test session. Hotter color means more respiration energy of the signal and colder less, represented in arbitrary units. During this session, P15 breathed following the metronome with a clear pattern, hotter in 7 b/m (RF). (RR interval: IBI).

As shown in Fig. 3 (bottom graph), a regular breathing rate generates a regular and periodic curve in the IBI, whereas a random breathing rate would not have created such a regular curve. Another way to represent this effect is using spectro-temporal analysis. Figure 3 (top graph) also represents the spectrum analysis as function of time (spectrogram) of breathing for each breathing rate (7, 6.5, 6, 5.5, 5 b/m) during the HRVB of a participant during the Test for the complete IBI series. The spectrogram was estimated using a short-time fourier transform (STFT)^29^ with a sliding window length of 120 s, with a resolution of 1.5 s. It can be seen that during this session, the participant breathed following the metronome.

Table 1 shows HRV parameters (means and SD) in Test and Retest for the two groups formed according to the RF change between the Test and the Retest: "RF Change" (n = 14) and “RF no-change” (n = 7). No statistically significant differences were found according to MANOVA. Likewise, no statistically significant differences are detected between the two groups (Mann–Whitney test) or between the two sessions, separately by groups or for the total (Wilcoxon test), applying non-parametric tests. However, the 14 participants who showed RF changes between test and retest showed lower IBImean values in both sessions than “RF no-change” group. In addition, these 14 participants showed a IBImean increasing between test and retest, while the other 7 participants who did not show changes in RF, showed a decrease in IBImean in the Retest. SDNN and RMSSD behave similarly (see Table 1). When analyzing the correlation independently for the two RF groups between HRV parameters (IBImean, SDNN and RMSSD) and RF value in each session, only a significant negative correlation was found for the “RF non-change” group between IBImean and RF value, both in the Test (rho = − 0.823; p = 0.023) and in the Retest (rho = − 0.805; p = 0.029). In contrast, “RF change” group shows no significant correlation in any session.

**Table 1:** HRV parameters [means (SD)] in Test and Retest for the two groups formed according to the RF change between the Test and the Retest: "RF Change" (n = 14), and “RF no-change” (n = 7).

Parameter	RF Group	Test	ReTest
IBImean	Change (n = 14)	753.6 (110.2)	771.6 (94.9)
	No-change (n = 7)	810.3 (98.9)	803.14 (109.6)
SDNN	Change (n = 14)	74.0 (38.4)	82.9 (38.9)
	No-change (n = 7)	92.3 (33.7)	88.1 (41.4)
RMSSD	Change (n = 14)	44.9 (29.5)	50.0 (30.6)
	No-change (n = 7)	50.1 (27.3)	46.0 (27.9)

### IBImean

**Figure 4:**
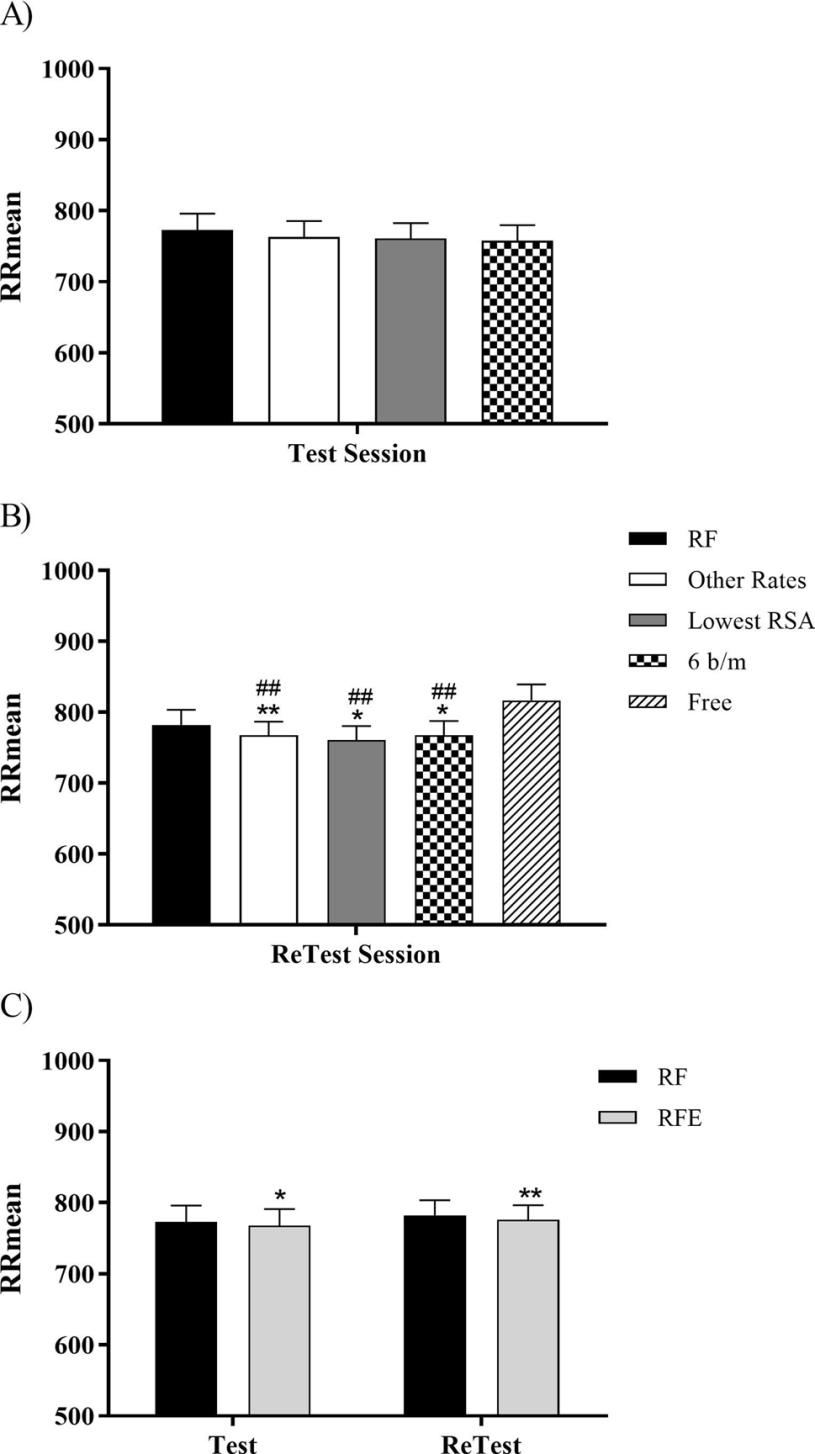
The mean interval time (ms) between heart beats (IBImean or RRmean) is represented for Test (**A**), ReTest (**B**) and RF-RFE comparison (**C**). “RF” represents the optimal resonance frequency, “Other Rates” represents the mean of the other breathing rates, “Lowest RSA” represents the lowest RSA amplitude, “6 b/m” represents the rate of 6 breathes per minute, “Free” represents the free slow breathing session only in retest, and “RFE” represents the expanded RF that consists on taking the IBImean of the nearest below and above rates from the RF. (Non-parametric Wilcoxon test has been performed when Friedman test showed significant differences.). * (p < 0.05) and ** (p < 0.01) mean differences with respect to RF in the same session. ^##^ (p < 0.01) means differences with respect to Free Respiration in the same session.

Figure 4 shows IBImean (mean interval time (ms) between consecutive heart beats) recorded in different sections or moments of the analysis. According to a Friedman test, there are no significant differences in IBImean in the Test session (Fig. 4A) comparing the different breathing rates (RF, mean of the other breathing rates, breathing rate with the lowest RSA and rate of 6 b/m). However, the Friedman test showed significant differences for IBImean between the breathing rates in Retest session. The Wilcoxon test for pairwise comparisons indicated that IBImean was slightly lower for the mean of other breathing rates, for the lowest RSA amplitude and for 6 b/m than for RF step (*p* < 0.05). IBImean showed no differences between free slow breathing and RF step, but these two steps showed higher IBImean values than all other breathing categories (*p* < 0.005) (Fig. 4B). Finally, IBImean was lower for RFE than for RF in both Test (*p* < 0.05) and Retest (*p* < 0.01) (Fig. 4C). No IBImean differences were found between Test and Retest sessions for RF and RFE rates.

### HRV time domain parameters

**Figure 5:**
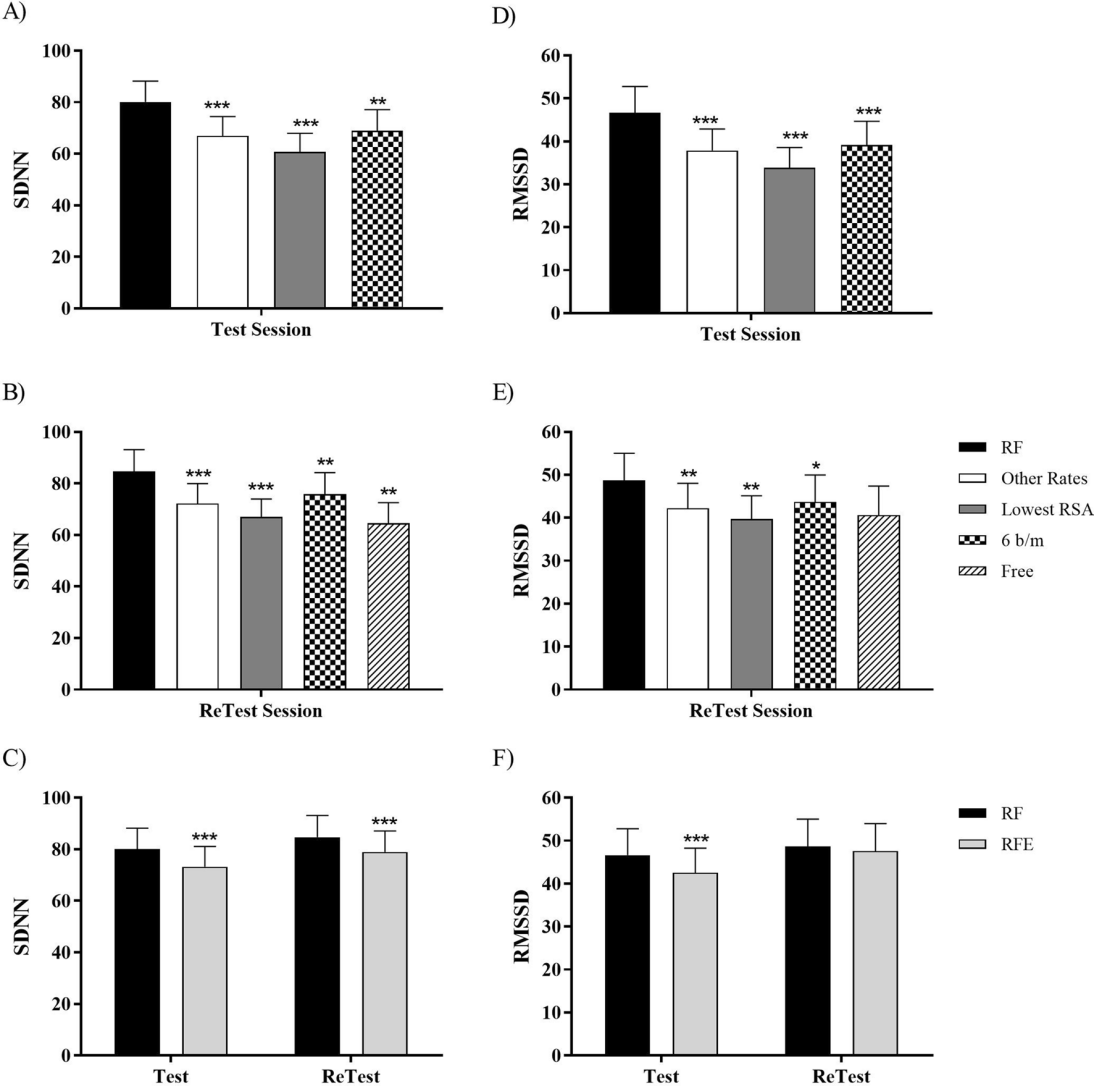
HRV time domain parameters: SDNN (**A**–**C**) and RMSDD (**D**–**F**) for Test (**A**,**D**), ReTest (**B**,**E**) and RF-RFE comparison (**C**,**F**). “RF” represents the optimal resonance frequency, “Other Rates” represents the mean of the other breathing rates, “Lowest RSA” represents the lowest RSA amplitude, “6 b/m” represents the rate of 6 breathes per minute, “Free” represents the free slow breathing session only in retest, and “RFE” represents the expanded RF that consists on taking the SDNN/RMSSD of the nearest below and above rates from the RF. Non-parametric Wilcoxon test has been performed. * (p < 0.05), ** (p < 0.01) and *** (p < 0.001) mean differences with respect to RF in the same session.

The effects of the different breathing rates on SDNN and RMSSD are represented in Fig. 5. In the Test session (Fig. 5A) as well as in the Retest session (Fig. 5B), SDNN was higher for RF than for all other breathing categories (*p* < 0.01) In particular, SDNN was higher for RF than for free slow breathing in retest session (*p* = 0.003). However, in this session SDNN shows no significant differences for free slow breathing than for the mean of other breathing rates, the lowest RSA amplitude and 6 b/m. SDNN was also higher for RF than for RFE in both sessions (*p* < 0.001; Fig. 5C), but no SDNN differences were found between Test and Retest sessions for RF. RMSSD was also higher for all other breathing categories compared to RF in both sessions (*p* < 0.05; Fig. 5D,E). However, RMSSD was not statistically different for free slow breathing than for all rates, including RF in the Retest session. Finally, RMSDD was higher for RF than for RFE only in Test session (*p* < 0.001) (Fig. 5F). No RMSSD differences were found between Test and Retest sessions for RF.

## Discussion

Results indicated that RF was not a constant cardiorespiratory parameter in the same individual, even in a short-term period (1 week). In addition, participants breathing at RF achieved the highest cardiac variability in time domain HRV parameters compared to other breathing rates and to free slow breathing. Therefore, these outcomes establish the recommendation to assess RF before starting each HRVB session in order to obtain the maximum benefit of this relaxation technique.

Control variables were easy to follow and check, thus it is also recommended to assess them in future studies.

The main result of this study was the discovery that RF was not stable between Test and Retest sessions. If we only look at statistical values of central tendency, we can observe as RF mean (6.1 b/m in Test, 5.9 in Retest) was similar and RF median (6 b/m) was equal in both sessions. However, the comparative box plots of RF individual values show a different distribution between Test and Retest (Fig. 2). It turns out that actually 14 out of 21 subjects had a different RF during the retest compared to test. We grouped these 14 participants as a "RF change” group, and the other 7 participants as a "RF no-change” group. The differences for IBImean values (Table 1) and for RF vs IBImean correlations between the two RF groups ("change" vs. "non-change") suggest that IBI could play a crucial role for the physiological determination of the RF. We also found consistency between IBImean, SDNN and RMSSD when showing increased cardiac variability in the Retest, although not significant, only for the group of participants who have shown changes in RF. In our case, we evaluated the control variables that may influence changes in cardiac variability between sessions. Thus, the study setup was similar in the test and the retest condition, and the questionnaires showed similar results. However, in most participants, the values of HRV-related parameters varied from mean value between two sessions conducted on different days, especially the mean value of IBI (IBImean). It could be that those participants who show a significant change in the mean value of IBI also show a change in the RF value between sessions. Anyways, these results need to be corroborated with a larger sample.

It is possible that the RF instability differences between the two groups are due to the different synchronization that the participants present between the cardiac and respiratory systems. As we have defined it, HRVB is based on breathing at an optimal rate (RF) corresponding to RSA. It is probable that RSA is mainly affected by breath frequency. And it may be possible to control the cardiovascular parameters by controlling respiratory rate and the number of heartbeats contained in the exhalation and inspiratory phase of the respiratory cycle^30^. But we have not analyzed this synchronization in our study, nor can we know if certain synchronization ratios may be influenced by the particular ages and genders of the participants, as noted in other study^31^.

We have already commented that in many investigations it has been assumed that RF is stable over time. But what would happen if it is not stable for the same participant between sessions held on different days? Our results indicate that RF changes for most participants between different sessions. According to Lehrer et al.^1^, it is considered that RF can change as a result of training. In the same publication they also state that RF can change over time within individuals, based on previous studies by his own group. However, in our study there was no such training and the changes observed in RF between sessions seem relevant. If these same results are confirmed in further research, it would indicate that breathing at RF that was not determined immediately before each breathing session makes no sense. Thus, because of the instability of RF, RF determined during HRVB sessions would not produce expected results during home practice.

In a recent publication, Shaffer et al.^32^ question how reliable RF assessment is, and highlight the lack of evidence on RF test–retest reliability. An RF instability similar to that of our study was previously found but using a different methodological approach. Thus, a feedback system that modulates breathing rate and HR during the breathing session was used after the first session^18^. Gross et al.^17^ also performed a test–retest as regards the RF, but they found an RF stability consisting of a range of 0.5 similar to the RFE variable. Finally, Hallman et al.^33^, following a similar approach as Lin et al.^18^, checked the RF for each session in a posteriori analysis and found stability between ten sessions. Therefore, our results confirmed the previous outcomes reported by Lin et al.^18^. On pooling participants, the RF was around 6 b/m (0.1 Hz), which is the most common rate. In addition, we demonstrated the relevance of increasing the range up to 7 b/m, since it was the optimal breathing rate for 13 participants in the Test or Retest sessions. However, we did not know whether any participant could have breathed at 4.5 b/m or even less. For future studies, it will be interesting to adapt the breathing ratios based on the type of sample^34^. Previous studies that controlled the mean of RF among participants found values between 5.5 and 6.5^33,35–41^. Breathing at a certain rhythm influences the heart rate pattern, as shown in Fig. 3. According to the creators of the HRVB method, the parasympathetic nervous system is enhanced through the vagus nerve breathing at RF/RSA, whereas the blood pressure drops as a consequence of the baroreflex stimulation. On the other hand, on slow breathing, but not following the RF, the RSA fluctuation may not be so obvious and their psychophysiological benefits, like increasing HRV, may not be obtained^3^.

The importance of breathing at the RF has been demonstrated in relation to HRV. As shown in Fig. 5, breathing at the RF increased both time domain parameters, SDNN, and RMSSD, compared to the other breathing rates. These results confirm the importance of assessing the RF before each session individually, because breathing at other rates were significantly worse than breathing at RF rate in terms of HRV. In addition, no significant differences were found between free slow breathing and the other breathing rates. As a healthy heart is not a metronome^21^, this increase of variability could mean an increase in cardiovascular and psychological health. According to some studies, whereas SDNN would be more influenced by low frequencies and by both sympathetic and parasympathetic nervous system, RMSSD would be the time domain parameter used to estimate the predominance of vagal activity and strongly correlates with the HF power parameter^21^. In addition, RMSSD is proportional to SD1 (a non-linear metric from the Poincaré Plot) that indicates the amount of variability in a short-term period^41^. Previous studies have shown that HRVB, assessing the individual RF only at the beginning of the intervention, enhanced HRV time domain parameters compared to controls^42^ and to baseline^35,36,43^. Lin et al.^18^, who also found RF changes over time, found that HRVB increased HRV parameters and baroreflex sensitivity, as well as reduced BP in pre-hypertension participants. On the other hand, there are also studies that find no significant differences or partial benefits of HRVB^6,37,44^. All of those investigations, however, did not assess RF before each session, which could explain the discrepancies found in the previous literature (Supplementary Information).

An alternative to search for the individual RF is to stablish a breathing rate of 6 b/m. In our study, SDNN and RMSSD showed significantly lower values at 6 b/m compared to RF (Fig. 5). Nevertheless, to pre-set a breathing rate of 6 b/m could not to be a bad option in case of not being able to assess RF before each HRVB session, as the overall mean of RF was 6. Previous studies also found positive effects of breathing at 6 b/m. For example, HRV parameters increased during the 10 min of breathing at 6 b/m in male students^45^; and RMSSD increased in a single breathing session at 6 b/m in adolescents with intellectual disability^46^. Nevertheless, there were no effects on applying a pre-set rate of 6 b/m. For example, a 3-day intervention of HRVB reduced anxiety, but increased time domain parameters of HRV, as well as the exercise and passive control groups did^47^. HRVB exerted no effect on time domain parameters of HRV in pain patients^48^, perhaps in this case because participants did not follow the target of 6 b/m. A rate of 5.5 b/m is another common and recommended breathing rate^20^. In fact, Lin et al.^49^ compared 5.5 b/m vs 6.0 b/m and found that breathing at 5.5 b/m with the same inhalation/exhalation (I:E) ratio significantly increased SDNN compared to 6.0 b/m at a different I:E ratio, but it was statistically equal to 6.0 b/m at the same I:E ratio; and, in all breathing rates, SDNN was higher than baseline. A progressive biofeedback system based on the RSA instead of a fix breathing rate is another alternative. This system works in real time, making users to follow the RSA or HR optimal wave for each moment. But despite all these studies, for now it is still unknown whether breathing at RF produce superior outcomes compared with 6-bpm or others slow paced breathings^32^. Equally, we have no evidence that RF breathing has better clinical outcomes than others slow paced breathings in treatment of most disorders^13^.

We also examined the proposal of an expanded RF (RFE) instead of a unique RF in order to try to obtain the same cardiovascular benefits with a reduction of the cost of assessing RF before each session. However, results indicated that RFE obtained worse results in SDNN and RMSSD than the RF (Fig. 5). This outcome was consistent with the previous study of Steffen et al.^16^. They found that breathing at RF + 1 b/m was not as positive as breathing at RF in frequency domain parameters of HRV, but not effects were found in time domain like in our study. We did not find statistically significant differences in the Test for IBI average between the different breathing categories, but we did find them in the Retest (Fig. 4). These results are in the same line already mentioned suggesting that the IBI average may be involved in RF stability.

On the other hand, the improvement on cardiac variability (HRV parameters) could be independent from the average pace of the heart beats. IBI average and HRV parameters are related by both mathematical and physiological models, through the autonomic balance^50,51^. In general, increased vagal activation causes major IBI and higher HRV values. However, HRV analysis has unique properties that make it a useful index for analysing the psychophysiological state. HRV, unlike IBI, allows the assessment of cardiovascular autonomic parameters that are under control of the sympathetic and the parasympathetic systems^22^. In certain situations IBI average and HRV parameters could not be as similar as expected, like it has been found in this study. In fact, in other breathing practices like Yoga, an increase of LF/HF ratio was correlated with a decrease of IBI^52^. The authors of that review agree that this a priori contradictory effect could be due to the levels of mental concentration. An active breathing as the one presented herein could implied heightened levels of attention, which increased the metabolic rate and the heart rate (reduction in IBI). In another study it was found that an increase in HRV parameters during slow-paced breathing was not mediated by changes in the mean cardiac vagal tone^53^. Lehrer et al.^13^, authors of HRVB method, have indicated in a recent systematic review that HRVB directly stimulates a variety of homeostatic reflexes such as the interaction between RSA and the baroreflex, in addition to stimulating parasympathetic activity^2,13^. This may be a probable reason why HRVB doesn’t decrease heart rate average.

Maybe the main limitation of this study is that the sample is small and not very heterogeneous. Larger sample studies are necessary to corroborate these initial findings as regards the instability of the RF and the importance of assessing individual RF to obtain cardiovascular benefits. Another limitation could be that in our study we determined RF only based on the maximum amplitude from the spectral analysis. To corroborate our results, it would be convenient to apply procedures that can determine comprehensively a more accurate RF by combining other HRV indicators. The estimation of the resonant frequency should represent the best convergence of different selection criteria^2^. In this regard, six criteria have been described as a strategy for identifying potential resonance frequencies: the greatest increases in phase synchrony, peak-trough amplitude, LF power, maximum LF amplitude peak, heart rate curve smoothness, and the fewest LF peaks. But researchers have not yet validated their weights, and this requires experimental confirmation^32^. The lack of control of the I:E ratio was another limitation of this and the most of studies; since, it has been proven to be as a relevant parameter of HRVB^49^. On the other hand, although in our study we instructed participants to breathe deeply, we did not objectively verify this. Our goal was not to check the possible effects of respiratory depth on HRV parameters. However, control over variables like ventilation, oxygen saturation, tidal volume or blood pressure could have given us information about differences between spontaneous and paced breathing^54^.

In conclusion, this study indicates that the resonance frequency value is not always stable over time and it suggests the relevance of assessing RF individually before each HRVB session. RF was not stable in the one-week test–retest protocol under the same methodological conditions. This instability could be related to the average of interbeat interval (IBI). We strongly recommend to check RF regularly during long-term interventions. We have also pointed out the importance of breathing at an individualized and momentary frequency of resonance to obtain the maximum benefits in terms of cardiac variability. We found that other breathing rates induced significantly lower cardiac variability and were no different from slow free breathing.

## Supplementary Information

Supplementary Information 1.

Supplementary Information 2.
