# Supplementary material for: Resonance frequency is not always stable over time and could be related to the inter-beat interval
Source: Sci Rep. 2021 Apr 16;11:8400. doi: 10.1038/s41598-021-87867-8 (PMC8052415; doi:10.1038/s41598-021-87867-8)
Supplement: Supplementary file 1 — Supplementary Information 1. [file 41598_2021_87867_MOESM1_ESM.docx]

**Resonance Frequency is not always stable over time and could be related to the inter-beat interval.**

Lluis Capdevila, Eva Parrado, Juan Ramos-Castro, Rafael Zapata-Lamana, and Jaume F Lalanza

**Appendix 1**: The Resonance Frequency (RF) for every participant and for test and retest sessions.

|  | Cohort | Test | Retest |
| --- | --- | --- | --- |
| Participant 1 | 1 | 7 | 5.5* |
| Participant 2 | 1 | 5.5 | 5.5 |
| Participant 3 | 1 | 7 | 6* |
| Participant 4 | 1 | 6.5 | 5* |
| Participant 5 | 1 | 5 | 6* |
| Participant 6 | 1 | 5.5 | 6* |
| Participant 7 | 1 | 5 | 5 |
| Participant 8 | 1 | 7 | 6* |
| Participant 9 | 1 | 6 | 6 |
| Participant 10 | 2 | 7 | 5.5* |
| Participant 11 | 2 | 7 | 7 |
| Participant 12 | 2 | 5 | 5 |
| Participant 13 | 2 | 5.5 | 7* |
| Participant 14 | 2 | 5.5 | 6* |
| Participant 15 | 2 | 5 | 5 |
| Participant 16 | 2 | 7 | 7 |
| Participant 17 | 2 | 5.5 | 7* |
| Participant 18 | 2 | 7 | 5.5* |
| Participant 19 | 2 | 7 | 6* |
| Participant 20 | 2 | 5.5 | 5* |
| Participant 21 | 2 | 7 | 6* |
| Mean (SD) | 1+2 | 6.1 (0.85) | 5.9 (0.69) |

* RF changes between Test and Retest
