# Supplementary material for: Resonance frequency is not always stable over time and could be related to the inter-beat interval
Source: Sci Rep. 2021 Apr 16;11:8400. doi: 10.1038/s41598-021-87867-8 (PMC8052415; doi:10.1038/s41598-021-87867-8)
Supplement: Supplementary file 2 — Supplementary Information 2. [file 41598_2021_87867_MOESM2_ESM.docx]

**Resonance Frequency is not always stable over time and could be related to the inter-beat interval.**

Lluis Capdevila, Eva Parrado, Juan Ramos-Castro, Rafael Zapata-Lamana, and Jaume F Lalanza

**Appendix 2:** Resonance Frequency (RF) based on the maximum amplitude of the RSA for each participant (Part.) and the RRmean, SDNN and RMSSD for each breathing rate at test (t) and retest (r) session.

|  |  | **Test** | | | | **ReTest** | | | |
| --- | --- | --- | --- | --- | --- | --- | --- | --- | --- |
|  | B. Rate | Max. A. RSA | RRmean  (ms) | SDNN  (ms) | RMSSD  (ms) | Max. A. RSA | RRmean  (ms) | SDNN  (ms) | RMSSD  (ms) |
| Part. 1 | 7 (t) | 35,52 | 868,07 | 134,98 | 105,56 | 27,57 | 761,98 | 127,00 | 86,77 |
|  | 6.5 | 32,24 | 816,99 | 117,76 | 80,17 | 26,96 | 756,84 | 119,14 | 75,94 |
|  | 6 | 27,44 | 751,58 | 115,39 | 61,36 | 31,86 | 767,29 | 140,16 | 96,45 |
|  | 5.5 (r) | 27,42 | 767,83 | 116,90 | 77,26 | 35,49 | 789,61 | 142,25 | 103,88 |
|  | 5 | 28,01 | 768,72 | 119,86 | 73,37 | 29,83 | 802,67 | 138,14 | 88,40 |
| Part. 2 | 7 | 26,08 | 802,72 | 90,93 | 58,42 | 25,76 | 799,64 | 89,24 | 62,15 |
|  | 6.5 | 26,20 | 809,74 | 89,16 | 52,55 | 27,89 | 777,23 | 93,69 | 52,24 |
|  | 6 | 26,36 | 808,89 | 91,64 | 54,32 | 24,30 | 830,18 | 95,94 | 62,01 |
|  | 5.5 (t-r) | 29,58 | 836,42 | 101,51 | 57,78 | 30,36 | 784,49 | 111,70 | 53,44 |
|  | 5 | 26,93 | 832,51 | 99,28 | 48,98 | 29,65 | 821,04 | 101,70 | 49,92 |
| Part. 3 | 7 (t) | 15,20 | 698,85 | 63,00 | 39,32 | 22,72 | 849,96 | 96,43 | 67,88 |
|  | 6.5 | 7,11 | 699,83 | 45,83 | 26,39 | 18,63 | 824,32 | 82,00 | 63,33 |
|  | 6 (r) | 11,49 | 706,53 | 57,75 | 34,46 | 26,34 | 895,37 | 102,38 | 66,09 |
|  | 5.5 | 12,78 | 758,50 | 51,77 | 33,38 | 22,22 | 866,24 | 88,47 | 54,72 |
|  | 5 | 9,82 | 739,15 | 52,19 | 24,74 | 22,50 | 861,94 | 98,03 | 52,66 |
| Part. 4 | 7 | 29,03 | 638,18 | 98,79 | 60,21 | 26,36 | 605,14 | 99,51 | 53,19 |
|  | 6.5 (t) | 32,05 | 656,50 | 107,50 | 68,16 | 28,48 | 606,10 | 98,68 | 53,26 |
|  | 6 | 30,07 | 661,29 | 112,97 | 72,03 | 33,28 | 630,70 | 113,96 | 64,51 |
|  | 5.5 | 29,72 | 637,08 | 125,35 | 74,77 | 33,84 | 643,90 | 112,43 | 62,82 |
|  | 5 (r) | 30,00 | 635,64 | 128,37 | 84,50 | 36,85 | 640,91 | 121,30 | 60,39 |
| Part. 5 | 7 | 29,27 | 939,18 | 114,13 | 84,36 | 41,87 | 972,83 | 144,22 | 111,78 |
|  | 6.5 | 31,09 | 906,69 | 112,68 | 69,32 | 38,39 | 961,02 | 136,74 | 102,69 |
|  | 6 (r) | 28,81 | 936,22 | 101,60 | 63,51 | 47,22 | 948,05 | 164,32 | 116,40 |
|  | 5.5 | 21,26 | 937,95 | 85,78 | 47,77 | 46,44 | 905,39 | 163,36 | 117,71 |
|  | 5 (t) | 33,86 | 929,47 | 127,04 | 73,11 | 39,14 | 968,93 | 151,73 | 103,60 |
| Part. 6 | 7 | 21,53 | 693,69 | 74,07 | 38,67 | 18,34 | 690,25 | 89,14 | 50,57 |
|  | 6.5 | 20,56 | 679,44 | 71,20 | 37,11 | 16,37 | 666,37 | 88,69 | 43,73 |
|  | 6 (r) | 21,47 | 682,13 | 76,01 | 39,40 | 25,54 | 701,78 | 97,69 | 64,06 |
|  | 5.5 (t) | 26,15 | 691,90 | 90,60 | 49,43 | 22,45 | 701,93 | 93,60 | 57,16 |
|  | 5 | 25,70 | 706,64 | 87,86 | 49,56 | 17,82 | 713,70 | 100,45 | 55,09 |
| Part. 7 | 7 | 19,55 | 924,30 | 75,10 | 44,80 | 21,18 | 901,80 | 75,93 | 46,63 |
|  | 6.5 | 16,53 | 918,91 | 73,13 | 38,26 | 25,34 | 918,36 | 121,58 | 64,10 |
|  | 6 | 31,10 | 926,03 | 129,61 | 92,25 | 25,90 | 928,20 | 103,81 | 57,43 |
|  | 5.5 | 39,15 | 940,15 | 135,16 | 94,99 | 32,27 | 969,79 | 109,73 | 67,27 |
|  | 5 (t-r) | 41,62 | 945,57 | 146,52 | 93,98 | 38,43 | 1005,74 | 152,72 | 90,41 |
| Part. 8 | 7 (t) | 34,61 | 794,79 | 112,91 | 70,78 | 21,33 | 659,32 | 74,05 | 35,78 |
|  | 6.5 | 23,81 | 795,30 | 109,64 | 58,66 | 16,67 | 657,08 | 70,03 | 32,66 |
|  | 6 (r) | 20,24 | 771,07 | 88,42 | 47,39 | 23,07 | 678,36 | 80,90 | 38,68 |
|  | 5.5 | 26,11 | 782,61 | 97,96 | 46,68 | 14,96 | 670,37 | 64,91 | 34,45 |
|  | 5 | 27,55 | 806,33 | 93,08 | 45,67 | 11,73 | 659,37 | 57,85 | 32,72 |
| Part. 9 | 7 | 21,81 | 741,18 | 100,47 | 54,76 | 23,56 | 747,39 | 91,88 | 53,29 |
|  | 6.5 | 21,40 | 739,64 | 94,32 | 55,12 | 19,09 | 732,63 | 94,90 | 67,22 |
|  | 6 (t-r) | 29,03 | 754,71 | 116,75 | 68,93 | 28,61 | 757,73 | 101,44 | 53,49 |
|  | 5.5 | 23,62 | 745,92 | 91,05 | 51,32 | 28,15 | 745,90 | 102,61 | 52,77 |
|  | 5 | 18,86 | 780,10 | 92,92 | 47,51 | 27,00 | 743,29 | 98,64 | 45,33 |
| Part. 10 | 7 (t) | 10,13 | 976,29 | 102,73 | 75,37 | 8,61 | 853,85 | 80,88 | 79,31 |
|  | 6.5 | 9,23 | 962,08 | 91,29 | 68,46 | 5,10 | 870,91 | 80,86 | 71,02 |
|  | 6 | 5,11 | 974,86 | 110,57 | 71,86 | 4,92 | 876,81 | 83,62 | 59,12 |
|  | 5.5 (r) | 8,08 | 1020,71 | 96,67 | 70,19 | 10,29 | 879,95 | 93,62 | 62,95 |
|  | 5 | 7,64 | 1020,07 | 108,48 | 64,87 | 9,50 | 872,43 | 101,22 | 71,77 |
| Part. 11 | 7 (t-r) | 6,05 | 638,75 | 59,28 | 25,67 | 2,53 | 639,82 | 34,84 | 12,25 |
|  | 6.5 | 4,11 | 613,37 | 33,60 | 14,68 | 1,41 | 618,81 | 19,21 | 6,58 |
|  | 6 | 2,72 | 643,36 | 23,58 | 11,14 | 1,50 | 647,76 | 17,96 | 7,09 |
|  | 5.5 | 3,99 | 652,17 | 28,73 | 11,15 | 1,15 | 678,92 | 20,91 | 9,68 |
|  | 5 | 1,38 | 689,17 | 26,07 | 8,09 | 1,40 | 676,87 | 23,74 | 9,94 |
| Part. 12 | 7 | 6,45 | 776,28 | 47,48 | 24,54 | 6,04 | 795,94 | 48,23 | 23,72 |
|  | 6.5 | 6,67 | 781,66 | 53,68 | 26,24 | 7,71 | 773,37 | 49,99 | 26,23 |
|  | 6 | 8,09 | 784,27 | 54,05 | 28,02 | 7,46 | 772,17 | 63,67 | 28,56 |
|  | 5.5 | 10,18 | 799,86 | 65,73 | 32,40 | 8,09 | 799,77 | 55,26 | 28,15 |
|  | 5 (t-r) | 10,34 | 825,91 | 70,76 | 30,14 | 9,58 | 802,29 | 68,97 | 32,04 |
| Part. 13 | 7 (r) | 6,43 | 802,99 | 50,22 | 29,02 | 7,76 | 855,57 | 64,94 | 41,20 |
|  | 6.5 | 5,27 | 806,10 | 44,46 | 21,28 | 5,19 | 830,17 | 51,30 | 25,24 |
|  | 6 | 4,84 | 794,29 | 42,71 | 19,75 | 4,25 | 824,09 | 44,77 | 22,31 |
|  | 5.5 (t) | 7,05 | 804,74 | 51,10 | 22,92 | 5,20 | 834,61 | 39,69 | 20,29 |
|  | 5 | 6,23 | 843,26 | 47,73 | 23,81 | 7,53 | 857,51 | 54,48 | 25,74 |
| Part. 14 | 7 | 2,59 | 590,73 | 23,77 | 10,39 | 7,09 | 701,55 | 50,98 | 32,66 |
|  | 6.5 | 2,93 | 591,21 | 24,47 | 10,17 | 6,72 | 672,57 | 49,56 | 27,47 |
|  | 6 (r) | 3,23 | 596,81 | 26,58 | 10,45 | 8,17 | 672,07 | 53,15 | 26,96 |
|  | 5.5 (t) | 4,13 | 589,94 | 28,15 | 11,20 | 7,60 | 689,42 | 51,64 | 25,77 |
|  | 5 | 1,77 | 593,10 | 21,15 | 8,35 | 6,13 | 679,61 | 46,62 | 22,67 |
| Part. 15 | 7 | 3,33 | 887,19 | 45,01 | 21,15 | 4,27 | 845,57 | 51,42 | 19,28 |
|  | 6.5 | 2,38 | 854,88 | 21,95 | 11,99 | 2,60 | 830,06 | 34,09 | 13,83 |
|  | 6 | 3,70 | 846,80 | 34,59 | 14,42 | 3,42 | 804,40 | 34,80 | 13,90 |
|  | 5.5 | 3,49 | 897,62 | 36,65 | 15,87 | 3,55 | 831,32 | 24,73 | 13,58 |
|  | 5 (t-r) | 4,44 | 888,84 | 53,03 | 17,12 | 4,70 | 843,75 | 44,06 | 16,23 |
| Part. 16 | 7 (t-r) | 14,57 | 780,97 | 96,78 | 57,35 | 16,01 | 787,81 | 102,74 | 65,65 |
|  | 6.5 | 13,02 | 744,88 | 89,65 | 45,99 | 15,90 | 772,14 | 101,67 | 59,90 |
|  | 6 | 13,65 | 745,35 | 91,98 | 44,11 | 13,08 | 721,16 | 86,95 | 43,60 |
|  | 5.5 | 13,20 | 736,60 | 87,21 | 37,75 | 11,59 | 715,25 | 76,16 | 32,90 |
|  | 5 | 13,84 | 744,47 | 88,28 | 40,19 | 12,57 | 756,03 | 83,32 | 38,37 |
| Part. 17 | 7 (r) | 2,74 | 714,18 | 29,97 | 14,82 | 3,86 | 713,19 | 41,06 | 24,49 |
|  | 6.5 | 3,04 | 700,85 | 24,23 | 15,12 | 2,81 | 685,56 | 28,06 | 17,28 |
|  | 6 | 2,15 | 699,52 | 19,76 | 12,94 | 2,09 | 674,96 | 26,68 | 11,77 |
|  | 5.5 (t) | 3,21 | 708,86 | 25,92 | 14,80 | 2,89 | 699,06 | 23,94 | 16,12 |
|  | 5 | 1,60 | 719,85 | 20,31 | 12,08 | 2,33 | 687,39 | 19,39 | 8,30 |
| Part. 18 | 7 (t) | 4,93 | 643,54 | 36,60 | 17,49 | 5,18 | 755,60 | 46,09 | 27,84 |
|  | 6.5 | 3,77 | 623,21 | 27,83 | 11,66 | 5,75 | 750,66 | 39,24 | 20,29 |
|  | 6 | 3,96 | 625,88 | 26,93 | 10,74 | 5,99 | 763,85 | 44,99 | 19,97 |
|  | 5.5 (r) | 4,35 | 636,99 | 31,26 | 10,54 | 6,70 | 782,95 | 44,95 | 21,30 |
|  | 5 | 3,92 | 642,28 | 28,80 | 10,14 | 6,11 | 780,03 | 48,02 | 19,57 |
| Part. 19 | 7 (t) | 10,41 | 740,20 | 75,00 | 41,92 | 7,39 | 698,10 | 60,02 | 30,35 |
|  | 6.5 | 9,88 | 723,82 | 71,40 | 37,69 | 7,46 | 704,96 | 56,50 | 27,96 |
|  | 6 (r) | 10,14 | 755,87 | 75,07 | 40,91 | 9,63 | 699,27 | 59,72 | 28,76 |
|  | 5.5 | 9,99 | 755,63 | 71,91 | 33,57 | 8,48 | 712,37 | 64,12 | 29,82 |
|  | 5 | 7,48 | 766,62 | 58,79 | 32,69 | 5,88 | 719,91 | 56,32 | 27,97 |
| Part. 20 | 7 | 3,59 | 689,73 | 25,18 | 13,70 | 1,92 | 714,95 | 20,08 | 9,96 |
|  | 6.5 | 3,25 | 688,31 | 25,96 | 12,07 | 1,62 | 722,02 | 27,20 | 11,49 |
|  | 6 | 1,84 | 699,13 | 15,72 | 7,72 | 2,04 | 728,49 | 27,00 | 11,41 |
|  | 5.5 (t) | 4,68 | 698,37 | 34,26 | 14,15 | 3,25 | 746,26 | 33,10 | 17,89 |
|  | 5 (r) | 2,11 | 708,40 | 26,02 | 10,94 | 5,28 | 740,46 | 48,95 | 20,78 |
| Part. 21 | 7 (t) | 5,94 | 750,47 | 44,74 | 25,15 | 6,53 | 813,60 | 54,37 | 30,65 |
|  | 6.5 | 4,42 | 728,69 | 31,62 | 17,36 | 5,28 | 769,02 | 44,67 | 21,87 |
|  | 6 (r) | 4,19 | 749,09 | 31,11 | 18,13 | 6,76 | 804,96 | 45,98 | 25,38 |
|  | 5.5 | 3,36 | 737,20 | 26,02 | 14,82 | 6,24 | 805,62 | 43,76 | 23,80 |
|  | 5 | 3,22 | 747,50 | 26,92 | 15,69 | 5,91 | 810,67 | 43,13 | 22,57 |

*t and r in brackets indicate the RF in test (t) and retest (r) sessions. Significant differences are not indicated in this table (*see Figures 4,5*).
